# Successful Treatment of Paradoxical Vocal Cord Motion with Sub-dissociative Dose Ketamine: Case Report

**DOI:** 10.5811/cpcem.24830

**Published:** 2025-04-07

**Authors:** Keaton Cameron-Burr, Elizabeth Terry-Kantor, Taneshia Wilson

**Affiliations:** Brown University, Department of Emergency Medicine, Providence, Rhode Island

**Keywords:** paradoxical vocal cord motion, ketamine, case report

## Abstract

**Introduction:**

Paradoxical vocal cord motion (PVCM) is a primarily neuropsychiatric condition that causes inappropriate adduction of the vocal cords during respiration. This condition is commonly misdiagnosed and treated as refractory asthma or upper airway obstruction requiring intensive care unit-level of care. Recent expert opinion suggests that ketamine administration may promote PVCM symptom resolution; however, this phenomenon has not yet been documented in the literature.

**Case Report:**

This is the case of a 23-year-old female who presented to the emergency department (ED) with acute PVCM exacerbation. After failing to respond to standard-of-care therapies including benzodiazepines, the patient was administered intravenous, sub-dissociative dose ketamine, which led to symptom resolution and discharge.

**Conclusion:**

Sub-dissociative dose ketamine may be a safe and effective therapy for PVCM exacerbations in the ED. In this report we explore the patient factors that likely mediated the clinical outcome in this case.

## INTRODUCTION

First documented by clinicians in the late 1800s in the setting of women’s “hysteria,” paradoxical vocal cord motion (PVCM) describes inappropriate adduction of the vocal cords during the respiratory cycle. Cases of PVCM can be divided into subtypes according to their pathophysiologic drivers. In primary PVCM, neuro-psychological pathology such as depression and anxiety drive symptom exacerbations. In secondary PVCM, neuromedical processes such as post-viral encephalopathy and gastroesophageal reflux trigger PVCM symptoms.^1^

Paradoxical vocal cord motion is commonly misdiagnosed as refractory asthma or upper airway obstruction, leading to inappropriate pharmacotherapy, intubation, and critical care utilization. Based on expert opinion, the authors of a 2017 review suggested that ketamine administration may be helpful in emergency department (ED) management of PVCM.^2^ The use of ketamine in ED management of PVCM has not previously been published. Here, we present a case of successful treatment of PVCM in the ED following administration of intravenous sub-dissociative dose ketamine. The antidepressant and synaptogenic effects of ketamine in combination with standard-of-care treatment likely mediated symptom resolution in this case.

## CASE REPORT

A 23-year-old female presented to the ED with a chief complaint of wheezing. Her past medical history was significant for PVCM, prior pulmonary embolism, tracheobronchomalacia, gastroesophageal reflux disease (GERD), depression, anxiety, and long-term sequelae of severe acute respiratory syndrome coronavirus-2 (SARS-CoV-2) infection. She endorsed central chest pain without pleurisy and denied recent illness, sick symptoms, and tobacco or vape use. The patient expressed that her current symptoms felt similar to prior PVCM exacerbations.

Six months prior to presentation, the patient was diagnosed with PVCM in the setting of SARS-CoV-2 infection. Since then, she had experienced worsening depression and anxiety and had frequent presentations to the ED with subjective respiratory distress resulting in multiple admissions to the intensive care unit (ICU). She was followed in the outpatient setting by an otolaryngologist and previously received botulinum toxin vocal cord injections with mild improvement in symptomatology. She was also in the process of seeking care from a gastroenterologist for severe gastroesophageal reflux disease.

On exam, the patient was afebrile and tachycardic to 106 beats per minute with a blood pressure of 140/90 millimeters of mercury. Her respiratory rate was 18 breaths per minute with an oxygen saturation of 98% on room air. The patient’s lungs were clear to auscultation. Intermittent grunting sounds were present on laryngeal auscultation, and the oropharynx was clear and without pharyngeal swelling. Despite her forced exhalation, the patient was able to speak in complete sentences and had normal work of breathing.

Laboratory evaluation was notable for a mildly elevated leukocyte count of 12.6 × 10^3^ cells per microliter (μL) (reference range: 4.0–11 × 10^3^ cells/μL). Other lab values, including a D-dimer, two high-sensitivity troponin levels, and a basic metabolic panel were within normal limits. A pregnancy test and a 22-pathogen respiratory viral panel were negative. Chest radiograph was without aberration, and a 12-lead electrocardiogram showed sinus tachycardia without evidence of acute ischemia. A diagnosis of PVCM exacerbation was made and nasopharyngoscopy deferred.

The patient was encouraged to breathe deeply and received 1 milligram (mg) of intravenous (IV) lorazepam without improvement. Given lack of response, the patient received a second dose of lorazepam, IV pantoprazole 40 mg, and oral solution containing 1% lidocaine, aluminum hydroxide, and magnesium hydroxide. Her symptoms persisted, after which IV ketamine 0.15 mg per kilogram diluted in 400 milliliters (mL) of normal saline was administered over 15 minutes. On patient re-evaluation 10 minutes after the initiation of ketamine infusion, mild clinical improvement was appreciated, with a decrease in severity of patient distress. On patient re-evaluation 30 minutes after the initiation of ketamine infusion, clinical resolution of PVCM exacerbation was noted. Ninety minutes following ketamine infusion, the patient expressed symptom resolution and the desire to be discharged to ED staff. She was observed for 120 minutes from the time of ketamine administration, demonstrating the ability to safely ambulate and a return to mental status baseline at which time she was discharged. She was counseled to follow up with her otolaryngologist, psychiatrist, and gastroenterologist and to return to the ED if she developed respiratory distress.

## DISCUSSION

Here, we present the first case report on the successful treatment of PVCM using sub-dissociative dose ketamine. This condition can be divided into categories based on underlying pathophysiology (Figure).

**CPC-EM Capsule**

What do we already know about this clinical entity?

*Paradoxical vocal cord motion (PVCM) is primarily driven by psychologic distress, although anatomic, neurologic, and medical factors may also contribute*.

What makes this presentation of disease reportable?

*This is the first reported case of emergency department (ED) management of PVCM using sub-dissociative dose ketamine*.

What is the major learning point?

*PVCM may be well controlled with sub-dissociative dose ketamine in the ED, particularly when symptoms are refractory to standard treatment*.

How might this improve emergency medicine practice?

*We were able to discharge the patient following ketamine administration, preventing misuse of critical care resources and limiting inappropriate medical therapy*.

Primary PVCM, which represents 75% of cases, is driven by neuropsychological pathology such as depression and post-traumatic stress disorder.^3^ Exacerbations are frequently precipitated by an increase in acute life stressors, and while many cases are attributed to airway obstruction, the majority of patients presenting to the ED with PVCM do not have respiratory failure.^2^ Secondary PVCM, which constitutes 25% of cases, results from a neurologic, respiratory, gastroesophageal, or other medical process.^3^

The patient presented here was likely experiencing a PVCM attack of mixed etiology. She had previously been diagnosed with depression and anxiety, esophageal reflux, and tracheobronchomalacia, all of which may contribute to the development of PVCM symptoms. Despite her history, her symptoms were refractory to topical anesthesia, acid suppression, and anxiolysis. Recent neurologic injury from infection with SARS-CoV-2 may have contributed to her symptom severity as well, as coronaviruses are well known to enter the cerebrospinal fluid, cause damage to brain tissue, and may precipitate or exacerbate psychiatric and functional neurological pathology.^4^,^5^ Both the patient and her family observed that her depression, anxiety, and PVCM symptoms worsened following her SARS-CoV-2 infection.[Fig f1-cpcem-9-169]

Depression, anxiety, and possibly post-viral neurologic injury were the likely acute and semi-acute drivers of PVCM in this case, with congenital tracheobronchomalacia likely conferring a baseline anatomic risk as well. A variety of therapies have previously been shown to be effective in decreasing acute PVCM symptomatology, including benzodiazepines and, in one case report, haloperidol.^6^,^7^ Symptoms may respond to acid suppression, upright positioning, and topical lidocaine, especially when tracheobronchomalacia is present. Heliox, a mixture of helium and oxygen, and non-invasive positive pressure ventilation may decrease symptoms by promoting laminar, unobstructed airflow.^2^,^8^ Botulinum toxin injection and superior laryngeal nerve block are attempted by otolaryngologists in refractory cases, and the most severe cases are managed with tracheostomy.^2^ However, unless patients experience symptom resolution and stability following a short treatment course, many of these strategies are insufficient to promote hospital discharge from the ED. In the case presented, treatment with benzodiazepines was attempted twice without symptom resolution. In addition, treatment for acid reflux and laryngeal irritation were ineffective. Intravenous sub-dissociative dose ketamine produced timely relief of the patient’s symptoms and allowed her to be discharged home.

Ketamine is a rapid acting antidepressant observed to be clinically effective within hours of administration, which increases synaptogenesis and neuronal growth factor release.^9^ Ketamine’s neuroprotective mechanism of action likely explains its clinical efficacy relative to benzodiazepines in this case. Benzodiazepines, while anxiolytic, are not antidepressants and do not promote neuroplasticity. In this patient with depression and possible recent neurologic injury from SARS-CoV-2 infection, ketamine likely offered the greater therapeutic benefit. Laryngospasm may rarely occur with ketamine administration, and this phenomenon is correlated with high-dose IV administration.^11^ Sub-dissociative dosing is important to minimize possible iatrogenic harm in this population.

Paradoxical vocal cord motion is associated with risk of misdiagnosis, iatrogenic harm, and inappropriate allocation of medical resources.^1^ Use of asthma medication in this group has been shown to be as high as 85%, and patients with primary PVCM account for approximately 10% of patients at specialized centers seeking treatment for refractory asthma.^2^,^11^ In addition, patients with PVCM may be admitted to the ICU for airway monitoring, resulting in high cost/low value use of critical care resources.^1^ In one study, as many as 28% of patients presenting to the ED with PVCM were subject to inappropriate intubation.^12^ Early identification of PVCM exacerbations vs asthma/other respiratory dysfunction in an emergency setting and management with ketamine could limit iatrogenic harm, systems waste, and improve patient outcomes.

## CONCLUSION

A young woman presented to the ED with a chief complaint of wheezing and was diagnosed with exacerbation of paradoxical vocal cord motion. We demonstrate the novel management of a case of PVCM using sub-dissociative dose ketamine allowing for discharge from the ED. Ketamine’s antidepressant neuroprotective effects likely mediated successful treatment of PVCM symptoms in the case presented. Ketamine may be valuable in achieving symptom resolution and discharge of patients with acute PVCM exacerbation presenting to the emergency department.

## Figures and Tables

**Figure f1-cpcem-9-169:**
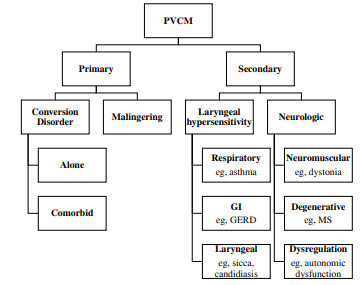
Subtypes of paradoxical vocal cord motion divided by primary driver. *PVCM*, paradoxical vocal cord motion; *GI*, gastrointestinal; *GERD*, gastroesophageal reflux disease; *MS*, multiple sclerosis.
